# Environmental Light Exposure Is Associated with Increased Body Mass in Children

**DOI:** 10.1371/journal.pone.0143578

**Published:** 2016-01-06

**Authors:** Cassandra L. Pattinson, Alicia C. Allan, Sally L. Staton, Karen J. Thorpe, Simon S. Smith

**Affiliations:** 1 Centre for Children’s Health Research, Institute for Health and Biomedical Innovation, Queensland University of Technology, Kelvin Grove, Queensland, Australia; 2 Centre for Accident Research and Road Safety – Queensland (CARRS-Q), Queensland University of Technology, Kelvin Grove, Queensland, Australia; University College London, UNITED KINGDOM

## Abstract

The timing, intensity, and duration of exposure to both artificial and natural light have acute metabolic and physiological effects in mammals. Recent research in human adults suggests exposure to moderate intensity light later in the day is concurrently associated with increased body mass; however, no studies have investigated the effect of light exposure on body mass in young children. We examined objectively measured light exposure and body mass of 48 preschool-aged children at baseline, and measured their body mass again 12 months later. At baseline, moderate intensity light exposure earlier in the day was associated with increased body mass index (BMI). Increased duration of light exposure at baseline predicted increased BMI 12-months later, even after controlling for baseline sleep duration, sleep timing, BMI, and activity. The findings identify that light exposure may be a contributor to the obesogenic environment during early childhood.

## Introduction

The contemporary child is exposed to greater daily duration and increased variation in intensity, temporal distribution, and spectra of *environmental light*, than children of any previous generation [1]. This is attributable to the use of artificial lighting, and has paralleled global increases in the incidence of obesity [1, 2]. Coupled with the known physiological impacts of light on human physiology, this raises a question; is light a factor in pediatric obesity?

It is estimated that 42 million children under the age of 5-years are classified as overweight or obese globally [3], including 23% of children in developed countries [4]. This is a significant clinical, public, and population health concern, as pediatric obesity is associated with a multitude of negative psychosocial and health sequela. Potential mechanisms that might be driving the global increase in obesity include increased calorie intake, decreased physical activity, and more recently, short sleep duration [5, 6], variable sleep timing [7], and gut flora [8]. However, attempts to address these factors have not, as yet, led to effective and sustained change in the prevalence and incidence of obesity [4]. Therefore, significant efforts are being made to identify modifiable factors that contribute to weight gain and that constitute the obesogenic environment. Recent evidence suggests that environmental light exposure may be one such factor.

Light is the principal cue for circadian entrainment in all species [9]. Circadian processes drive physiological and behavioral mechanisms including sleep-wake cycles [10], regulation of metabolism [11–13], emotion [14], and body mass [15–17]. Through the adoption and use of artificial lighting, humans have constructed a photoperiod that is malleable, creating an environment of relatively dim days and bright nights [1, 18]. Manipulation of the timing, intensity, and duration of light exposure to suit contemporary lifestyles has occurred with limited consideration of its effects on health, behavioral, and environmental outcomes. An understanding of these effects is only now beginning to emerge [1, 14, 18, 19].

Animal studies indicate that the timing and intensity of light exposure is critical for metabolic functioning and weight status. Rodents exposed to continuous white light, even at low levels, exhibited symptoms of metabolic syndrome, increased adiposity, glucose intolerance [20, 21], and reduced sympathetic activity in brown adipose tissue [22], independent of their caloric intake and locomotor activity. Many of these symptoms are abolished when regular light-dark cycles are reinstated [23]. Furthermore, studies of the natural environment indicate that increased artificial light at night, both through direct illumination (e.g. structural, security, street, and advertising lighting) and skyglow, affect the reproductive, migrative, and daily movement behaviors of multiple plant and animal populations [18, 24–26]. The cost of these changes are not yet fully understood. In adult humans, morning bright light treatment has been shown to reduce body fat and appetite [27, 28], improve mood [27], and modulate concentrations of the appetite regulating hormones; leptin and ghrelin [29]. Commensurately, recent evidence shows that exposure to light of moderate intensity (~500 lux) earlier in the day is associated with lower body mass, independent of sleep timing, total sleep duration, and activity in adults [17]. Taken together, these data indicate that the timing, duration and intensity of light exposure has a potent role in metabolic and physiological functioning. Early childhood is a pivotal time in the establishment of lifelong growth and adiposity trajectories [30]. However, to date, no studies have examined the effect of habitual light exposure on body mass in children.

The present study investigated the relationship between timing, duration, and intensity of light exposure and weight status of healthy, free-living children aged 3 to 5 years, both concurrently and longitudinally. Standardized independent measurements of *body mass index (BMI*; kg/cm^2^) were taken for all participating children at both time points. BMI measurements were transformed into age- and sex-specific BMI z scores for each child [31]. It was hypothesized that, independent of sleep midpoint (used as a proxy for sleep timing and circadian phase; [32]), sleep duration and activity, timing and intensity of light exposure (earlier in the day) would be associated with lower concurrent BMI z score. Further, it was hypothesized that timing of light exposure at baseline, would be associated with BMI z score 12-months later.

## Materials and Methods

### Ethics Statement

The study protocol was approved by Queensland University of Technology’s Human Research Ethics Committee. Written informed consent was provided by directors, teachers and the legal guardians of the children. Children gave their assent to participate.

### Study Design

Initially, 62 healthy pre-school children (32 Males (51.6%); *M* = 56.51 months, *SD* = 5.94, and Age Range: 39.0–74.0 months) were recruited for the 12 month study, from six long-day child care services in Brisbane, a capital city in subtropical Australia. Participating child care services were recruited from a pool of 118 services participating in a pre-existing study [33]. All services were located in high socio-economic status (SES) areas according to postcode (SEIFA [34]) and were randomly selected to be approached to take part in the study. Lower SES is associated with a range of sociodemographic factors which may impact upon child health and development [35–37], as such services in high SES areas were specifically targeted in the study design to control for some of these variations. Within each childcare service, one room catering for children within the preschool age range (3–5 years) was targeted for recruitment. All children attending the target rooms were invited to participate in the study. To avoid school holiday periods, the 14^th^ of December, 2012 (final date of the school term in Queensland, Australia) was the predefined study endpoint.

At baseline participating families were sent a 14-day sleep diary, parent survey and Actiwatch 2 (MiniMitter Phillips) device. Actigraphs were worn by the study child on the non-dominant wrist for 14 days. The sleep diary was completed concurrently by parents who were asked to record their child’s sleep and wake times, napping behaviour and any instances which the actigraph was removed from the child’s wrist. The parent survey included demographic information and an 8-item Food Frequency Questionnaire (FFQ), which asked parents to indicate, “In the last 24 hours, how often has the study child had the following foods?” with the following trichotomous responses available “Not at all,” “Once,” or “More than once” [38]. Within the 14-day testing period, researchers visited each participating pre-school classrooms on the same designated testing day each week (i.e. Tuesday, Wednesday, or Thursday). Researchers observed the childcare routine and environment, including sleep practices and behaviours, and on one visit measured each participant’s height and weight. The baseline measurements were conducted in the Australian spring/summer between October and December, 2012. During this period, in Brisbane, Queensland (study location) the average sunrise occurred at 4:55am and sunset occurred at 6:17pm.

Participating families were then contacted to participate again 12 months later (*follow-up*). Accordingly, the follow-up period was between October and December, 2013. All participating families were sent a parent survey and a researcher visited the family to collect the child’s height and weight measurements.

At baseline, complete data was obtained for 49 (79.03%) children. One participant was excluded due to insufficient actigraphy data (< 2 days), giving a final sample of 48 children at baseline. Of the 48 children who completed the baseline measurements, 9 children did not complete the follow-up BMI measurements, giving an attrition rate of 18.75%. No differences were found between those participants who did and did not complete the study at either time point, for gender, age or BMI.

### Measurement of light, activity and sleep

The Actiwatch devices measured children’s motor activity (range: 0.5–2G) and white light luminance exposure (range: 5–100,000 lux) in 1 minute epochs for 14 days. The Actiwatch 2 has been calibrated to the international standard ISO-10526 (CIE-S-005) and has been shown to measure illuminance of multiple white light sources in agreement with a National Institute of Standards in Technology (NIST)-traceable photometer [39]. Rest/sleep intervals were assessed by parent reported sleep diaries and wrist actigraphy, using Actiware 5.2 software (Phillips Respironics, Bend, Oregon 97701 USA). Sleep onset, offset and duration were determined using the parent-reported sleep diary in conjunction with the actigraphy data. Sleep onset was determined by using the exact diary time indicated by the parent; unless the time indicated fell on an epoch determined as “sleep” (S) by actigraphy then, sleep onset was operationalized as the last “wake” (W) epoch before the first 3 consecutive S epochs. Sleep offset was determined by using the exact diary time indicated by the parent; unless the time indicated fell on an epoch determined as S then, the time was extended until the first W epoch, after the last 5 consecutive S epochs. Data were cleaned using the Actiware 5.2 software which involved excluding periods in which the parent indicated that the actigraph was removed from the child’s wrist. Total sleep duration was calculated based on the mean duration of all sleep periods (day and night) over the 14-day period. Sleep midpoint was calculated based on the average of sleep onset and offset for the 14-day period. Sleep midpoint was used as a proxy for circadian phase [32]. For a conservative estimate of children’s motor activity and light exposure, any extended inactive periods (>5mins), not recorded by the parents in the sleep diary, were excluded.

### Light and activity analysis

**Fig 1:**
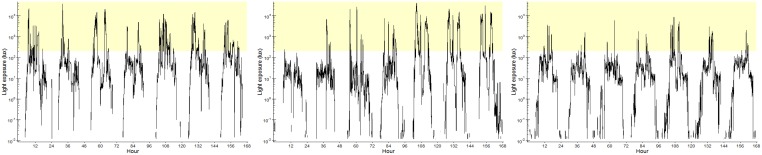
Smoothed 7-day light exposure plots from three individual participants. Light exposure data (measured in lux) were smoothed and shown on a logarithmic scale for three representative children across 7 measurement days (plotted in hours). The horizontal yellow shaded area represents a threshold of ≥200 lux. Points where lux is registered as zero are not shown, as the log of zero is not defined.

Intensity, duration and timing of exposure to light were determined by using a similar methodology to that described by Reid and colleagues [17]. Light and activity data were collected and exported from Actiware 5.2 software in .csv format at a 1-minute (epoch) resolution. Data was imported into *RProject 2*.*11*.*1*. and smoothed using a 5-minute rolling average (to account for the finer measurement window used compared to Reid and colleagues) [17], and then aggregated over 24-hours for each participant. Any 24-hour periods with greater than 4 hours of excluded data were considered invalid and subsequently excluded from further analyses. These aggregate data allowed the calculation of time above threshold (TAT), and mean light timing above threshold (MLiT) [17]. TAT is the average daily number of minutes (epochs) spent above a given lux threshold. This captures both, intensity and duration of light exposure. TAT intensity thresholds ranged from 10 to 3000 lux. MLiT [17] describes the daily distribution of light exposure. Calculation of MLiT incorporates intensity (lux threshold), duration (number of minutes above a threshold), and timing of exposure (clock time of each minute above the threshold; 10 to 3000 lux). The formula used to calculate MLIT was produced by Reid and colleagues [17], however in this study j (minute of day) = 1, …, 1440, as light exposure (lux) was measured at a resolution of 1 minute epochs for 24 hours (24 x 60 = 1440mins). Also, k (day) = 1, …, 14, as the children in this study wore the Actiwatch for 14 days. To illustrate, throughout 24-hours across a 14 day period, a MLiT^200^ of 721minutes indicates that the child’s light exposure above 200 lux was, on average, centred around 12pm (or the 721^st^ minute in the 1440 minute day from 12am). Representative examples of individual light profiles of participating children are illustrated in Fig 1. The measure of activity used in this analysis was the mean of each epoch of activity over 24 hours, across the 14 days of recording.

### BMI analysis

At both baseline and follow-up, height and weight were measured by trained researchers using calibrated stadiometers (SECA Leicester Portable Height Measure) and floor scales (HD-316, Wedderburn Scales; Tanita Corporation, Tokyo, Japan) with subjects dressed in light clothing, and without shoes. Children were measured twice and if measurements differed (weight >0.1kg; height >0.5cm) a third measurement was taken by the researcher. The mean of the measurements were used. Due to the high proportion of this sample being breast-fed at some point during infancy (88.4%) and no significant developmental delays identified, the WHO growth charts were utilised to calculate BMI and growth trajectories of the children [31]. BMI measurements were transformed into sex- and age-specific *z* scores using the WHO Anthro version 3.2.2 and AnthroPlus version 1.0.4.

### Statistical analysis

All analyses were conducted using SPSS v. 22.0.0.0. Sensitivity analyses were conducted to examine if any light variables were associated with BMI z score, both at baseline and follow-up. Consistent with the procedures used by Reid et al. [17], the light exposure variables with the highest correlation with BMI z score were then used in all subsequent analyses. Bivariate correlations (Tables A and B in S1 File) were run to examine the association between BMI z score at baseline and at follow-up with sleep midpoint, sleep duration, activity, diet variables, and the 24hr light variables identified in sensitivity analysis: TAT and MLiT. Sleep midpoint was not normally distributed so a log-linear transformation was conducted. Subsequently, all analyses shown include the log transformed sleep midpoint variable. Nutrition variables were available for a subset of children (n = 42). There was limited variability in parent reporting of nutritional intake. Furthermore, none of the nutritional intake items correlated with either BMI z score (baseline/follow-up) or the light variables. As such the nutritional items were not included within the final regression analyses. Multivariable linear regression models were then used to assess the relationships between BMI z score at baseline with activity, sleep midpoint, and sleep duration and the TAT and MLiT thresholds identified through bivariate correlations. To assess the relationship between light at baseline and BMI z score at follow-up another multivariable linear regression was conducted, adjusting for baseline measures of BMI z score, activity, sleep midpoint and sleep duration. Significance levels are indicated with asterisks: *p < 0.05; **p < 0.01; ***p < 0.001.

## Results

### Participant demographics

Participant demographic, BMI, BMI z score, sleep, activity and light characteristics for both baseline and follow-up are described in Table 1. At baseline, the average age of participating children was 4.76 years (SD = 4.94 months), with 52.1% of the sample being female. According to WHO percentiles 97.9% of participants were classified as within the healthy weight range, with one participant being classified as overweight/obese [31]. None of the participating children were classified as underweight, at either time point, in this study. Parents of the participating children predominantly identified themselves as Australian (67%). Please refer to Table C in S1 File for further information regarding identified ethnic group. Valid actigraphy recording ranged from 3–13 days (M = 10.7 days). The average sleep onset time was 20:37 (SD = 00:39), sleep offset was 06:03 (SD = 00:36), sleep midpoint was 01:19 (SD = 00:34), and total sleep duration was 9.64 hours (SD = 31.19 minutes). MLiT^200^ centered on 12:37 (SD = 00:36) with a range from 11:10 to 14:28. The average duration of time that participating children spent in light above the 200 lux threshold was 3.43 hours (SD = 7.06 minutes). In comparison, children spent 64 minutes (SD = 25.22 mins) above the 2500 lux threshold. At follow-up, the mean age of participants was 5.74 years (SD = 5.12 months) and 51.3% of the sample were female. The majority of children (92.3%) were classified as normal weight, two children (5.1%) classified as overweight and one child (2.6%) classified as obese [31].

**Table 1:** Participant demographic, sleep, activity, and light characteristics at baseline and follow-up.

Variable	N	Mean (SD)
**Child Demographic Variables**		
*Baseline*	**48**	
Age (months)		57.06 (4.94); range 45.90–64.66
Sex		25 F; 23 M
BMI		15.45 (1.10); range 13.61–18.79
BMI z—score		0.09 (.73)
*Follow-up*	**39**	
Age (months)		68.87 (5.12); range 56.77–76.88
Sex		20 F; 19 M
BMI		15.63 (1.12); range 14.02–20.08
BMI z—score		0.20 (.68)
**Actigraphy Variables**	**48**	
Days Actigraph Recorded		10.7 (2.03)
Activity (Mean Activity Count)		400.87 (58.25)
Sleep Midpoint (hh:mm)		01:19 (00:34)
Sleep Onset (hh:mm)		20:37 (00:39)
Sleep Offset (hh:mm)		06:03 (00:36)
Sleep Duration (minutes)		578.37 (31.19)
Mean TAT 10 LUX (minutes)		612.69 (67.24)
Mean TAT 100 LUX (minutes)		287.48 (67.16)
Mean TAT 500 LUX (minutes)		152.41 (38.39)
Mean TAT 1000 LUX (minutes)		117.72 (33.41)
Mean TAT 2500 LUX (minutes)		64.08 (25.22)
Mean TAT 3000 LUX (minutes)		54.06 (22.80)
MLiT 10 LUX (hh:mm)		12:32 (00:25)
MLiT 100 LUX (hh:mm)		12:28 (00:30)
MLiT 200 LUX (hh:mm)		12:37 (00:36)
MLiT 500 LUX (hh:mm)		12:38 (00:43)
MLiT 1000 LUX (hh:mm)		12:32 (00:45)
MLiT 3000 LUX (hh:mm)		12:06 (00:52)

### Light exposure profiles and baseline BMI z score

**Fig 2:**
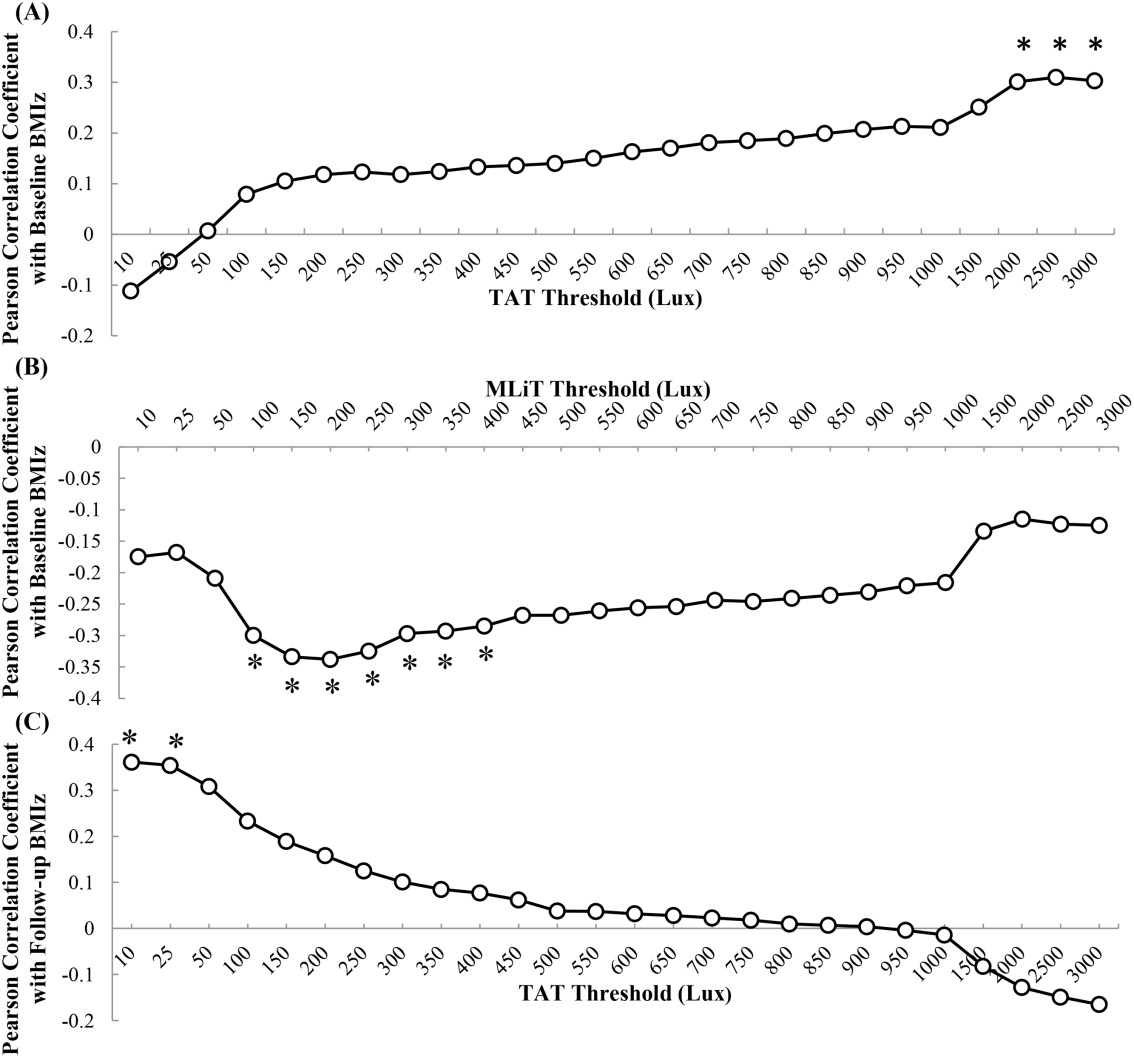
Sensitivity Analyses showing Pearson correlations between BMI z score and a range of MLiT and TAT Light Thresholds (lux) at baseline and follow-up. * indicates statistically significant at *p* < .05. **(A)** TAT thresholds of 2000–3000 lux were significantly associated with BMI *z* score at baseline, with TAT^2500^ having the strongest association (*N* = 48). **(B)** MLiT thresholds of 100–400 lux were significantly associated with BMI *z* score at baseline, with the strongest association at MLiT^200^ (*N* = 48). **(C)** TAT thresholds of 10–25 lux were significantly associated with follow-up BMI *z* score, with the strongest association at TAT^10^ (*N* = 39).

**Fig 3:**
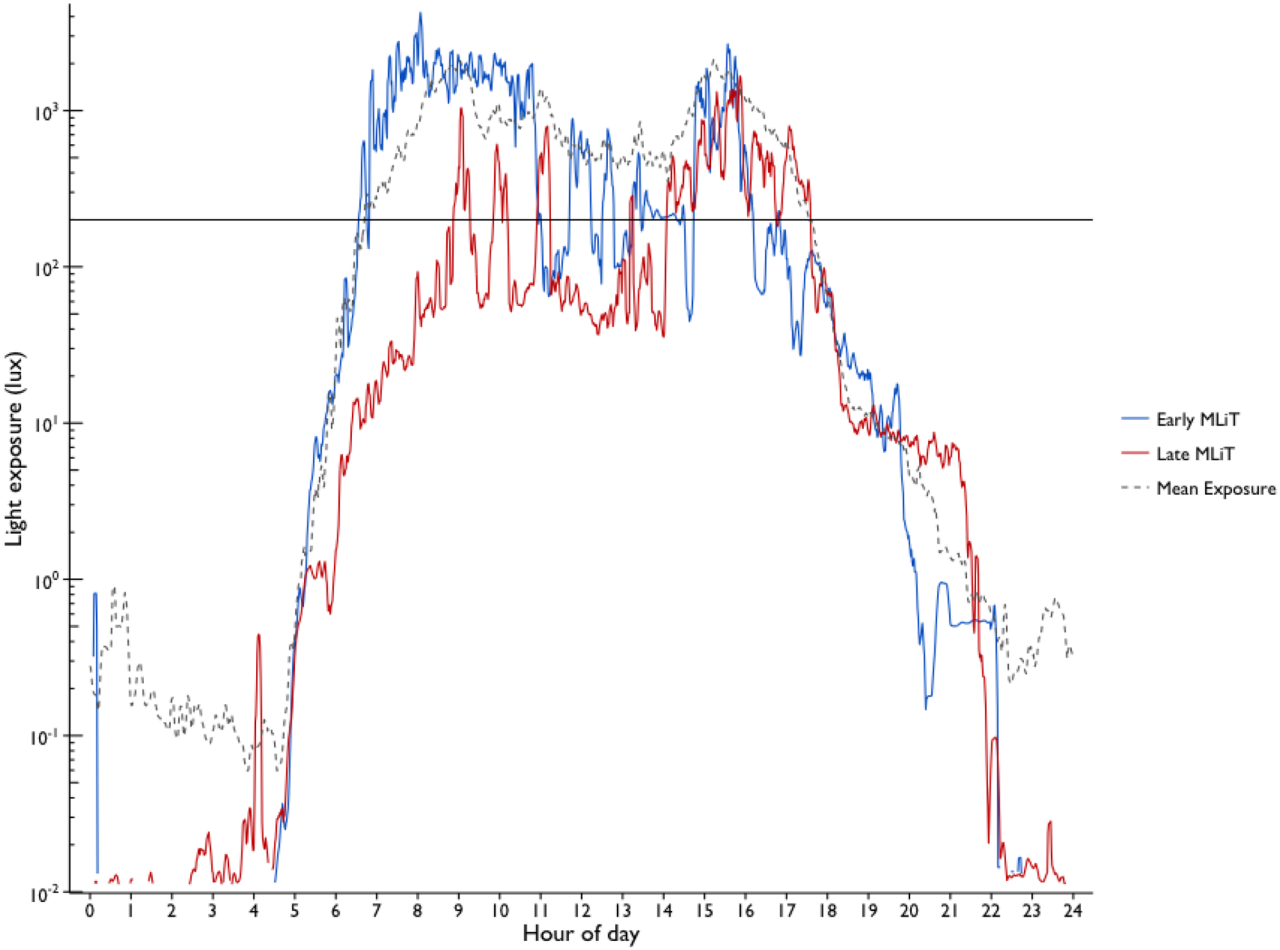
Representative light exposure profiles (log linear lux) for two individual participants with “Early” and “Late” light exposure. The black dashed line depicts the mean light exposure of all participants across the recording period (*N* = 48); the blue line depicts the light exposure of a participant classified as having an “Early MLiT^200^”; the red line depicts the light exposure of a participant classified as having a “Late MLiT^200^”. The horizontal line represents the 200 lux threshold.

To determine which 24-hour light variables (MLiT/TAT), across the thresholds of 10–3000 lux had the strongest association with baseline BMI *z* score, sensitivity analyses were conducted (Fig 2A and 2B). TAT^2500^ was identified as having the strongest association with baseline BMI *z* score. This association was positive, indicating that longer daily duration of light exposure above a threshold of 2500 lux was associated with higher BMI. This illuminance level would be equivalent to outdoor lighting on an overcast day [40, 41]. MLiT was also significantly associated with baseline BMI *z* score, with the strongest association occurring at MLiT^200^. Earlier light exposure above a threshold of 200 lux (MLiT^200^) was associated with higher BMI. Representations of early and later MLiT^200^ are illustrated in Fig 3. Illumination of 200 lux is approximate to a typical home living room or kitchen [42].

To determine the effect of duration and timing of light exposure on BMI z score, a hierarchical multivariable linear regression analysis was performed. Specifically, we examined the effect of TAT^2500^ and MLiT^200^ on BMI z score, after adjusting for activity, total sleep duration, and sleep midpoint (Table 2). The full model accounted for 27.3% of the variance in baseline BMI z-score (*F*_5,42_ = 3.153, *p* = .017, r^2^ = 0.273). In this model, later sleep midpoint was associated with increased BMI z score (*β* = .363, *p* = .020). Although TAT^2500^ was correlated with BMI z score, it was not a significant independent predictor when entered into the model. However, MLiT^200^ did have a significant, independent effect on BMI z score (*β* = -.419, *p* = .01). This result indicates that earlier exposure to moderate intensity light is associated with increased concurrent BMI z score in preschool children, independent of activity, total sleep duration, and sleep midpoint.

**Table 2:** Linear regression models predicting BMI *z* score at baseline and follow-up.

**(a)** Baseline BMI *z* score (*N* = 48)				
			***95% CI (b)***	
**Predictors**	***b* (*SE*)**	***β***	***Lower***	***Upper***
Activity (M)	.00 (.00)	-.01	-.00	.00
Sleep Midpoint^a^	1.36 (.57)	.36*	.22	2.50
Total Sleep Duration	.00 (.00)	.08	-.01	.01
TAT^2500^	.01 (.00)	.24	-.00	.02
MLiT^200^	-.01 (.00)	-.42*	-.02	-.00
**(b)** Follow-up BMI *z* score (*N* = 39)				
			***95% CI (b)***	
**Predictors**	***b* (*SE*)**	***β***	***Lower***	***Upper***
Baseline BMI *z* score	-3.69 (2.47)	.62***	.39	.87
Activity (M)	.00 (.00)	.02	-.00	.00
Sleep Midpoint^a^	.64 (.42)	.18	-.22	1.50
Total Sleep Duration	-.00 (.00)	-.08	-.01	.00
TAT^10^	.00 (.00)	.40**	.00	.01

*p < .05,

**p < .01,

***p < .001.

^a^Sleep Midpoint has been log transformed

### Light exposure at baseline predicts 12-month follow-up BMI z score

After examining the association between light exposure and BMI *z* score at baseline, we wanted to determine if baseline light exposure would predict BMI *z* score 12-months later. We conducted a sensitivity analysis to establish if any of the light variables at baseline had an association with BMI *z* score at follow-up, across each threshold (10–3000 lux). No baseline MLiT variables were associated with BMI *z* scores at follow-up. However, both TAT^10^ and TAT^25^ had a positive association with BMI *z* score at follow-up (Fig 2C), with the strongest association found for TAT^10^. Illumination of 10 lux is approximate to a candlelit room.

To test the hypothesis that baseline light exposure predicts BMI *z* score at follow-up (*N* = 39 children), a hierarchical multivariable regression analysis was conducted. This model was adjusted for baseline measurements of BMI *z* score, activity, sleep midpoint and total sleep duration (Table 2). The model significantly predicted a striking 59% of the variance in BMI *z* score at follow-up (*F*_5,34_ = 9.501, *p* < .001). Even after adjusting for baseline BMI *z*-score (*β* = .621, *p* < .001), TAT^10^ remained a significant and independent predictor (*β* = .400, *p* = .002) of BMI *z* score 12-months later. This result indicates that longer daily duration of light exposure greater than 10 lux at baseline is associated with increased BMI 12-months later, independent of baseline BMI, activity, sleep midpoint and total sleep duration.

## Discussion

This study is the first to investigate the relationship between the timing, duration, and intensity of light exposure and the body mass of young children. We found that daily environmental light exposure had a significant association with the children’s body mass, both concurrently and longitudinally. Our findings are consistent with those from studies conducted with animal models, and in adult humans, which indicate that variations in light exposure may influence body mass [17, 20–22, 27]. In the current study, earlier exposure to moderate levels of light was associated with higher concurrent BMI. In clinical terms, for every hour earlier that MLiT^200^ occurred during the day, there was a .6 unit increase in BMI. While this degree of body mass gain may seem modest, it could indicate an early deviation in a lifelong body mass trajectory. The direction of this relationship contrasts with those reported for adults, where earlier light exposure was found to be associated with decreased body mass [17]. The difference in the direction of these findings may reflect variations in biological timing and threshold of exposure at which light exerts an influence on physiological processes in young children. Consistent with this interpretation, a recent study indicated that adolescents have a heightened sensitivity to light exposure when compared to older adults [43]. Whilst the timing of light exposure at baseline was not predictive of BMI 12-months later, the duration of light exposure was. Specifically, longer duration of total light exposure at baseline was predictive of higher BMI at follow-up. The increased use of electronic equipment such as night lights, tablets, mobile phones, and televisions has been well-documented for children 3–5 years [44, 45]. The current result may help us to better understand findings of an association between this increased duration of screen use and light in the bedroom and body mass in children.

Our findings provide evidence consistent with profound metabolic and physiological effects of light on the human body [18, 19, 46–49]. These results are especially striking when we consider that BMI in the first five years of life is predictive of life-long body mass trajectories [30]. Unlike activity, dietary intake, and sleep duration, light exposure is easily and directly manipulated; literally through the flick of the switch. The current ubiquitous social, industrial, and culturally driven manipulation of our environmental light may impact on body mass through three very broad mechanisms that warrant exploration. Firstly, increased light duration may provide insufficient dark, and insufficient metabolic ‘down time’, for normal recuperative processes to occur. Indeed, depending on geographical location, skyglow and other artificial light at night sources are increasing at rates of up to 20% per year [50]. Children are increasingly exposed to broader spectral signatures and more diverse intensity profiles of light [51]. Secondly, chronically increased daily light duration may provide a biological signal analogous to endless summer days, with the potential to amplify any seasonally-driven metabolic processes, such as body mass acquisition [52, 53]. Alternatively, a child’s initial light state may promote some mediating phenomena such as problematic behavior, physiological or metabolic changes, which in turn, promote changes in BMI. One example of light states interacting with physiological behavior is in the case of sleep. Multiple studies document an association between short sleep duration and variability in sleep timing with increased body mass in pediatric populations [5–7]. Thus, a confounding relationship between sleep and light exposure is expected as sleep timing and duration likely to influence the timing and duration of light exposure. In this study sleep duration was not associated with either BMI or light exposure variables at either time point. Although surprising, this finding is consistent with some research conducted in the early childhood period [54, 55]. Furthermore, the null findings may also be explained by our use of ambulatory recording versus parent report methods used commonly in research reporting an association between sleep and body mass in children (see review [56]). However, it is noted that sleep midpoint was associated with timing of light exposure in this study. Further, in our model, later sleep midpoint was a significant independent predictor of increased BMI z-score. This indicates that timing of sleep and light exposure may be interacting to influence body mass of children.

There are limitations to our study. We have not measured the spectral signatures to which children are exposed, instead measuring light objectively in ambient lux (lumens/m^2^, weighted to human perception of brightness) [57]. Throughout the day, wavelength composition varies and studies have shown that spectral variations have very distinct impacts on different circadian, behavioral and physiological responses [58]. As such, it is recommended that future studies use devices that measure spectral power distribution, such as spectroradiometers [59]. Direct measurements of circadian phase and metabolic hormones were not determined in this study. Future work should include measurement of circadian phase and metabolic hormone variation of children to provide tests of the direct or indirect path of associations found between light and body mass. For example, timing of light exposure has been shown to affect expression of melatonin and shift circadian phase [59, 60], which in turn impacts on hormones such as insulin [12, 61]. Additionally, the light intensities shown to be significantly associated with body mass in this study need to be confirmed in a larger cohort of children. It is noted that in this study, body mass was treated as a continuum, with only a small number of children classified in the clinical range for overweight and obesity. As such future research could investigate this association using children in the clinical range for overweight and obesity. Although BMI has been shown to have good agreement with body composition in children [62], future studies could consider the use of other estimates of adiposity including; skin fold thickness, waist circumference, or dual energy x-ray. Further research is needed to address these issues as well as the mechanisms responsible for the association between light exposure and body mass in children.

We live in a society of relatively dim days and bright nights [1, 16]. The findings of this study suggest biologically inappropriately timed light exposure and ‘longer’ light periods, may be problematic for body mass of children. If light is in fact a meaningful and distinct contributor to body mass and weight gain, then quantification of light exposure could be included in clinical assessment protocols, and even used routinely in pediatric assessments concerned with incipient obesity. Furthermore, clinical prescription of ‘dark time', analogous to current light therapies, could restitute a state of shorter, brighter days and longer dark nights, with resultant increases in the amplitude of a child’s natural circadian rhythm. Indeed, inexpensive consumer-grade wearables already collect similar data to that provided by actigraphy, and individual tracking of habitual activity, sleep-wake patterns, and light exposure, is already possible. The rapid acceptance and uptake of these devices increases the potential for future effective and well-evaluated public health interventions around light exposure. Likewise, ‘smart house’ applications already allow control of artificial lighting in the home, school, and childcare environments, and provide another potential point for intervention with public health implications. By customizing our light environment, we have launched a global naturalistic experiment, the effects of which are only just beginning to emerge. Our data provides an impetus to investigate environmental light as a factor in the obesogenic environment during human development. This may reveal new targets for pediatric obesity intervention and prevention.

## Supporting Information

S1 File Table A. Bi-variate correlations between Baseline BMI z-score (BMIz), TAT, MLiT, sleep, and activity (*N* = 48). Table B. Bi-variate correlations between Follow-up BMI z score (BMIz) and Baseline BMI z score, TAT, sleep, and activity variables (*N* = 39). Table C. Proportion of parents in each identified ethnic group (*N* = 42).
(DOCX)
