# Supplementary material for: Environmental Light Exposure Is Associated with Increased Body Mass in Children
Source: PLoS One. 2016 Jan 6;11(1):e0143578. doi: 10.1371/journal.pone.0143578 (PMC4711797; doi:10.1371/journal.pone.0143578)
Supplement: S1 File — Table A. Bi-variate correlations between Baseline BMI z-score (BMIz), TAT, MLiT, sleep, and activity (N = 48). Table B. Bi-variate correlations between Follow-up BMI z score (BMIz) and Baseline BMI z score, TAT, sleep, and activity variables (N = 39). Table C. Proportion of parents in each identified ethnic group (N = 42). (DOCX) [file pone.0143578.s001.docx]

**S1 File. Supporting Information**

**Table A. Bivariate correlations between baseline variables (*N* = 48).**

|  |  | 1 | 2 | 3 | 4 | 5 | 6 |
| --- | --- | --- | --- | --- | --- | --- | --- |
| 1 | BMIz at Baseline | - | .31* | -.34* | .13 | .09 | -.10 |
| 2 | TAT^2500^ |  | - | -.36* | -.23 | .01 | -.01 |
| 3 | MLiT^200^ |  |  | - | .45** | .10 | .08 |
| 4 | Sleep Midpoint^a^ |  |  |  | - | .15 | -.13 |
| 5 | Sleep Duration |  |  |  |  | - | -.14 |
| 6 | Activity |  |  |  |  |  | - |

Note: TAT^2500^ is Time above threshold of 2500 lux; MLiT^200^ is Mean Light above Threshold of 200lux

*p < .05, **p < .01

^a^Sleep Midpoint has been log transformed

**Table B. Bivariate correlations between follow-up and baseline measures (*N =* 39).**

|  |  | 1 | 2 | 3 | 4 | 5 | 6 |
| --- | --- | --- | --- | --- | --- | --- | --- |
| 1 | BMIz at Follow-up | - | .65*** | .36* | .21 | -.07 | -.04 |
| 2 | BMIz at Baseline |  | - | -.00 | .22 | .06 | -.16 |
| 3 | TAT^10^ |  |  | - | -.27 | -.10 | .10 |
| 4 | Sleep Midpoint^a^ |  |  |  | - | .04 | -.05 |
| 5 | Sleep Duration |  |  |  |  | - | -.12 |
| 6 | Activity |  |  |  |  |  | - |

Note: TAT^2500^ is Time above threshold of 2500 lux; MLiT^200^ is Mean Light above Threshold of 200lux

*p < .05, ***p < .001

^a^Sleep Midpoint has been log transformed

**Table C. Proportion of parents in each identified ethnic group (*N =* 42).**

|  | Proportion of Parents (%) |
| --- | --- |
| Oceania | 66.7 |
| North-East Asian | 4.8 |
| North African and Middle Eastern | 2.4 |
| Southern and Central Asian | 2.4 |
| North-West European | 7.1 |
| Southern and Eastern European | 4.8 |
| No Specific Ethnic Identification | 11.9 |

Note: Ethnic group classification was identified in accordance to the Australian Bureau of Statistics - Australian Standard Classification of Cultural and Ethnic Groups (ASCCEG), 2011^[[1]](#footnote-1)^.

1. Australian Bureau of Statistics. 1249.0 - Australian Standard Classification of Cultural and Ethnic Groups (ASCCEG). 2011. Available online at: <http://www.abs.gov.au/ausstats/abs@.nsf/Latestproducts/1249.0Main%20Features12011?opendocument&tabname=Summary&prodno=1249.0&issue=2011&num=&view>= [↑](#footnote-ref-1)
